# Can digital breast tomosynthesis offer an alternative to MRI in preoperative imaging of lobular carcinoma?

**DOI:** 10.1186/bcr2977

**Published:** 2011-11-04

**Authors:** PD Wall, JC Morel, R Wasan, D Evans, C Peacock, MJM Michell

**Affiliations:** 1 Kings College Hospital NHS Trust, London, UK; 2 South East London BSP, London, UK

## Introduction

Digital breast tomosynthesis (DBT) is an emerging breast imaging technique which we have shown in our institution to more accurately size tumours when compared with 2D mammography. In this study we have looked at the accuracy of DBT in the sizing of invasive lobular carcinoma (ILC) when compared with MRI, the current imaging technique of choice.

## Methods

All clients diagnosed with ILC were identified between December 2008 and March 2010. Of these clients, those who had undergone imaging with both MRI and DBT were extracted. Tumour diameter in the single largest dimension was recorded on DBT by two consultant radiologists. MRI and pathological tumours sizes were extracted from the notes. The agreement between the two imaging modalities and pathology was analysed using the methods described by Bland-Altman. Pearson correlation coefficients were calculated.

## Results

Twenty-three cases of lobular carcinoma were identified with MRI and DBT imaging. Pearson correlation coefficients for DBT with pathology *r *= 0.73, MRI with pathology *r *= 0.86, MRI with DBT *r *= 0.81. See Table 1.

**Table 1:** 

	DBT-pathology	MRI-pathology
Upper limit of agreement (mm)	31 (15 to 47)	25 (12 to 38)
Mean difference (mm)	-11 (-20 to -1)	-9 (-16 to -2)
Lower limit of agreement (mm)	-52 (-68 to -36)	-43 (-56 to -30)

95% CI presented in parentheses.

## Conclusion

The study shows that MRI is superior to DBT in predicting the histological size of lobular carcinoma although there is disparity with both techniques. With the advent of contrast-enhanced 2D mammography, we hope for the future development of contrast-enhanced DBT to give us a technique that will be comparable with MRI.

